# Evaluation of the vitreous microbial contamination rate in office-based three-port microincision vitrectomy surgery using Retrector technology

**DOI:** 10.1186/1471-2415-14-58

**Published:** 2014-05-01

**Authors:** Flavio A Rezende, Cynthia X Qian, Przemyslaw Sapieha

**Affiliations:** 1 Department of Ophthalmology, University of Montreal, Montreal, Canada; 2 Department of Ophthalmology, Pontifícia Universidade Católica, Rio de Janeiro, Brazil; 3 Retina Service, Department of Ophthalmology, Massachusetts Eye and Ear Infirmary, Harvard Medical School, Boston, USA; 4 Department of Ophthalmology, Hôpital Maisonneuve-Rosemont, 5415, boulevard de l’Assomption, Montréal, Québec H1T 2 M4, Canada

## Abstract

**Background:**

To perform a microbiological contamination analysis of the vitreous during office-based micro-incision vitrectomy surgery (MIVS) assessing whether the bacteria detected correlated with patient's ocular conjunctival flora.

**Methods:**

This is a prospective, interventional, nonrandomized case series of patients undergoing office-based MIVS, anti-VEGF, and dexamethasone intravitreal injections (triple therapy) for the treatment of wet age-related macular degeneration (AMD) and diabetic macular edema (DME).

All patients were operated at a small procedure room in an ambulatory clinic of the Department of Ophthalmology, University of Montreal, Quebec, Canada. Conjunctival samples were done before placing the sclerotomies. The MIVS was done with a 23-gauge retractable vitrector, a 27-gauge infusion line, and a 29-gauge chandelier. Undiluted and diluted vitreous were collected for aerobic, anaerobic and fungal cultures. Outcomes measured were bacterial species identification within samples collected from the conjunctiva and the vitreous.

**Results:**

Thirty-seven patients (37 eyes) were recruited and completed over 17 months of follow-up. Twenty-eight had wet AMD and nine had DME. There were 13 men and 24 women, with a mean age of 78 years. Eighteen patients (46%) had culture positive conjunctival flora. Twenty-six bacterial colonies were tabulated in total from the conjunctival swabs. All bacteria detected were gram-positive bacteria (100%), most commonly: Staphylococcus epidermitis in 11 (42%) and Corynebacterium sp. in 6 (23%). Only 1/18 patients had more than 3 species isolated, 6/18 patients had 2 species and 11/18 patients had 1 species identified on the conjunctival swab. Only 1 of the 37 undiluted midvitreous samples was culture positive, equating to a contamination rate of 2.7%. None of the diluted vitreous samples were culture positive. All cultures were negative for fungus. No serious postoperative complications occurred, including bacterial endophthalmitis, choroidal detachment, and retinal detachment.

**Conclusion:**

This preliminary study of office-based MIVS gives us insights on the ocular surface microbial profile and vitreous contamination rate of performing such procedures outside the OR-controlled environment. Our initial results seem to indicate that there is little risk of bacterial translocation and contamination from the conjunctiva into the vitreous. Therefore, if endophthalmitis occurs post-operatively, the source may likely arise after the procedure. Larger studies are needed to confirm our data.

## Background

The recent technological innovations in continually refining and decreasing gauge size for vitreoretinal surgeries have further ignited interest in the development of office-based, portable microincision vitrectomy surgery (MIVS). Indeed, the concept of bringing vitrectomy surgery out of the operating room (OR) is not new. Starting in the early 1980s, case series on the successful use of portable vitrectors in rural, non-OR settings have been reported [1,2]. One of the latest commercial models is a cannula-less 23-ga needle with an incorporated 25-ga vitrector called the Retrector whose indications have evolved from a simple tap and inject to numerous diagnostic and therapeutic functions [3-5].

With the introduction of a new surgical technique, increased risks of complications and endophthalmitis are always a major concern [6,7]. One of the main suspected routes of contamination is through the transconjunctival pathway, in which insertion of surgical instruments directly through conjunctiva may inadvertently track ocular surface organisms into the vitreous [6-9].

In this prospective study, we sought to assess the rate of conjunctival bacterial translocation and vitreous contamination following office-based three-port vitrectomies performed using the Retrector portable vitrector. We also evaluated whether the vitreous isolates correlated to the species identified from the same patients’ conjunctiva.

## Methods

### Surgical method and sample collection

**Figure 1:**
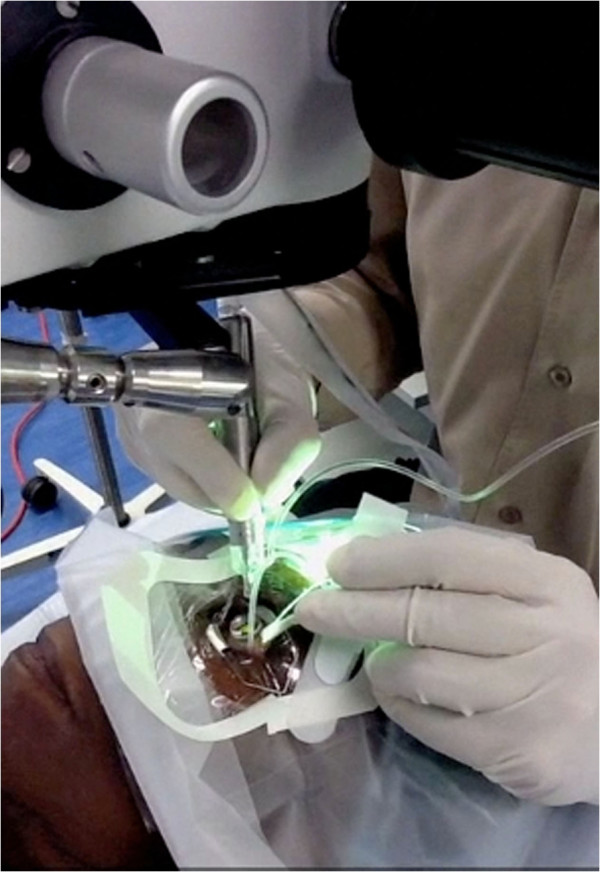
**Office-based vitrectomy set-up.** All procedures were performed in an in-clinic non-sterile non-positive pressure procedure room. Note that the surgeon is gloved and masked but not gowned. The instruments were handled in a sterile manner.

All patients were followed by a single retina specialist (FAR) and had been previously diagnosed with exudative age-related macular degeneration (AMD) or diabetic macular edema (DME). They were recruited from a pilot study on vitreous lipidomics measurements and quantification of intravitreal levels of different cytokines (Study ID: HMR-10059, http://www.clinicaltrials.gov), depending on the presence or absence of daily oral omega-3 supplementation received [Rezende, FA, Lapalme, E, Qian, CX, et al. Omega-3 supplementation influences vitreal levels of Vascular Endothelial Growth Factor in Exudative Age-Related Macular Degeneration. Paper presented at: ARVO Annual Meeting, May 8 2012, Ft Lauderdale]. Those who had received a minimum of 2 months of omega-3 supplementation prior to intervention were compared to case-matched patients who had received no supplements. For cytokines levels and lipidomics profiling, all patients underwent a one-time vitreous biopsy where undiluted vitreous samples were collected at the time of anti-VEGF (bevacizumab 1.25 mg/0.05 ml) and dexamethasone injections using a portable office-based vitrectomy device (Retrector, Insight Instruments, Inc, Stuart, FL) [4,10-12]. Half of each vitreous sample was sent for cytokines and lipidomics analysis whereas the other half was sent along with conjunctival samples for microbial analysis. Patients were excluded if they had evidence of local or systemic infections. The entire procedure was performed as an outpatient procedure in the minor procedure room of the ophthalmology ambulatory center at Maisonneuve-Rosemont Hospital. One single surgeon performed all surgeries using a mask and sterile gloves, as would be performed for a routine anti-VEGF injection (Figure 1). All instruments were opened and handled in a sterile manner. The study conforms to the tenets of the declaration Helsinki and was approved by the Institutional Review Board of the Maisonneuve-Rosemont Hospital affiliated with the University of Montreal.

### Initial conjunctival sampling

After written informed consent was obtained, the first conjunctival specimen sampling was performed prior to any anaesthetic or topical drops. Calcium alginate swabs were passed several times over the bulbar conjunctiva and then plated unto anaerobic and aerobic sleep blood agar, chocolate agar, Sabouraud agar and an enriched brain-heart infusion broth. The fornix was not sampled. Care was also taken not to let the swab come in contact with the lashes or conjunctival areas other than those that would lie directly over where the 3 ports were placed. The patients then received topical anaesthetic drops followed by a peribulbar injection of 2% lidocaine without epinephrine.

### Vitrectomy

**Figure 2:**
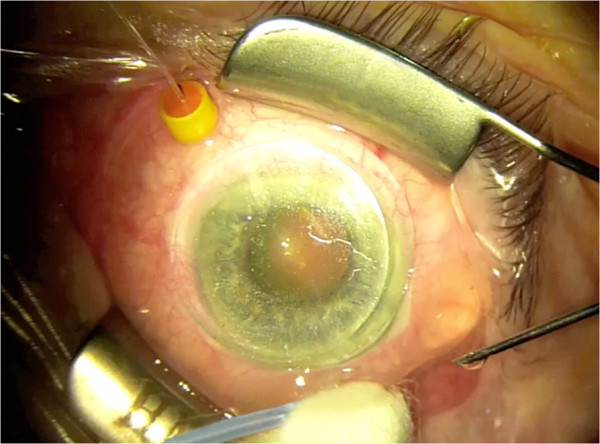
Set-up for office-based 3-port MIVS; superotemporally, inserted Retrector, inferotemporally self-retaining 27-ga infusion line and superonasally, 29-ga self-retaining chandelier illumination system.

All procedures were performed in an in-clinic non-sterile non-positive pressure procedure room. 5% povidone-iodine was used to clean the periocular skin and topical instillation into the eye and within the cul-de-sac was left in place for 5 minutes. The surgeon was gloved and masked but not gowned. The instruments were handled in a sterile manner. Patients were then draped in a standard sterile manner with placement of a lid speculum. A 27-ga self-retaining line (Insight Instruments, Stuart, FL) for balanced salt solution (BSS) infusion was first placed, followed by a 29-ga chandelier placement connected to a mercury vapor light source (Synergetics, O’Fallon, Mo). The surgical view during the procedure was provided through a surgical operative microscope and a Volk contact lens (Volk direct image 1.5x magnifying disposable vitrectomy lens, OH, USA). The vitrectomy was performed using a 25-ga sutureless Retrector system (Insight Instruments, Stuart, FL) in all patients. The model used in the study is a portable, battery-powered system with a maximum cut rate of 600 cpm and features a single-use retractable sheathed guillotine cutter (25-ga) within an in-built needle (23-ga). The needle was introduced bevel down through displaced conjunctiva in an oblique one-plane tunnel into the vitreous cavity 3-4 mm from the limbus (Figure 2) [3-5]. With the exception of the portable vitrector motor handpiece, which was placed within a sterile plastic cover when in use, all other instruments used were sterile and disposable.

Two other specimens were obtained from the vitreous cavity at the beginning and the end of vitrectomy. The first undiluted sample was aspirated using a sterile 1.0 ml syringe connected to the aspiration line of the portable vitrector. A minimum of 0.5 ml of undiluted vitreous fluid was cut and removed from the mid-vitreous through controlled manual aspiration, all the while visually monitoring for and avoiding globe collapse.

At the end of the vitrectomy, a second 10 ml sample of vitreous diluted through continuous BSS infusion was again manually collected in through the vitrector aspiration line. Both vitreous samples were promptly capped after collection and sent to the microbiology department at the end of the case. 0.8 mg (0.2 ml) of preservative-free dexamethasone and 2.5 mg (0.1 ml) of bevacizumab were then delivered on separate 30-ga needles into the eye through the 29-ga trocar after chandelier removal. The intraocular pressure (IOP) was digitally assessed and, if necessary, the remaining volume deficit was compensated by BSS. The integrity of the sutureless wounds was verified. If a leak or vitreous wick was noted, vitreous was removed with the vitrector and thermocauterization was applied to coapt the conjunctiva overlying the sclerotomy site. None of the sclerotomies needed to be sutured. All patients were seen at 24 hours, 1 week and 1 month postoperatively for follow-up.

### Bacterial cultures

Conjunctival samples and both undiluted and dilute vitreous samples were directly transported after collection to the Department of Microbiology of the same institution for further analysis with a delay <1 h after collection. The unplated vitreous samples were cultured under aseptic techniques unto anaerobic and aerobic agars. Incubation was performed at 37°C for 48 hours aerobically in an atmosphere of 7% CO_2_ and then anaerobically. Sabouraud agar at 25°C was used to enhance fungal isolation. Bacterial samples were kept for at least 1 week while fungal cultures were held for 1 month. Quantitative data were expressed as mean ± SD. Associations between microbial data and clinical findings that may predispose to bacterial presence was expressed as an odds-ratio. (SPSS for Mac; SPSS, Inc, Chicago, IL). Statistical significance was defined as *P* < .05.

## Results

A total of 37 consecutive eyes (37 patients) were recruited to the study between June 2011 and end of 2012. There were 13 men and 24 women. The mean age of patients was 77.9 ± 7.4 years and the mean follow-up time since vitrectomy was 16.9 ± 2.0 months. Five patients were on daily glaucoma medication drops, which were continued as usual prior to their visit. None of the patients wore contact lenses or used topical antibiotics in the 4 weeks prior to sampling. All patients continued to receive regular anti-VEGF treatments if their AMD or DME were deemed active after the office-based MIVS (Table 1).

**Table 1:** Patient characteristics

**Parameters**	**N**	**%**
No eyes/patients	37	
- Left eyes	12	32%
- Right eyes	25	68%
No. Men/women		
- Men	13	35%
- Women	24	65%
Age		
- Mean ± SD	77.9 ± 7.4 years	
- Range	46-97 years	
Follow-up		
- Mean ± SD	16.8 ± 2.0 months	
- Range	6-19 months	
Diagnosis		
- Exudative AMD	28	76%
- DME	9	24%
Phakic status		
- Phakic	16	43%
- Pseudophakic	21	57%
- Aphakic	0	0%
Medical conditions*		
- Glaucoma	5	14%
- Hypertension	17	46%
- Diabetes	14	38%
- Hyperlipidemia	8	22%
- Hypothyroidism	5	14%
- CAD	4	11%
- Asthma	4	11%
- Osteoporosis	2	5%

CAD: coronary artery disease; SD: standard deviation; DME: diabetic macular edema.

*Medical conditions are non-additive and one patient may manifest several of the most common comorbid conditions.

In all, 18 patients (46%) had culture positive conjunctival flora for a total of 26 colonies from eight different species of bacteria. All of these isolates (100%) were gram-positive and identified as organisms found as part of the normal ocular flora [13]. The most commonly found species were *Staphylococcus epidermitis* (11/26; 42%) and *Corynebacterium sp* (6/26; 23%). Only 1/18 patients had ≥3 species isolated (*Staphylococcus epidermitis, Staphylococcus warneri, Streptococcus mitis*), 6/18 patients had 2 species and 11/18 patients had 1 species identified on the conjunctival swab (Table 2).

**Table 2:** Most commonly isolated bacterial species from conjunctival surface

**Bacterium**	**Conjunctival flora N (%)**
Staphylococcus epidermitis	11 (42%)
Corynebacterium sp.	6 (23%)
Propionibacterium sp.	3 (12%)
Staphylococcus auricularis	2 (8%)
Staphylococcus aureus	1 (4%)
Corynebacterium macginleyi	1 (4%)
Staphylococcus warneri	1 (4%)
Streptococcus mitis	1 (4%)

sp: species.

Only 1 of the 37 undiluted midvitreous samples was culture positive, equating to a contamination rate of 2.7%. The bacterium isolated was *Propionibacterium sp.* This patient’s conjunctiva also grew *Propionibacterium sp*. The dilute vitreous sample from this patient was culture negative. None of the 37 dilute midvitreous samples were culture positive and there was no fungal growth in any sample.

Although there was a slightly higher rate of male gender (OR 1.01, 95% CI 0.26-3.92, P = 0.99), diabetes mellitus (OR 1.30, 95% CI 0.34-4.93, P = 0.39) and hypertension (OR 1.92, 95% CI 0.52-7.00, P = 0.32) amongst those with detection of positive conjunctival flora cultures, the values were not statistically significant.

All patients did well after samples collection. Only one patient in the cohort experienced asymptomatic hypotony with an intraocular pressure of 6 on post-operative day 1. This transient phenomenon self-resolved completely on the next follow-up one week later. None developed serious postoperative complications, including bacterial endophthalmitis, choroidal detachment, and retinal detachment.

## Discussion

The use of modern office-based vitrectomy has greatly evolved since it was first introduced [3-5,10-12,14]. Newer models have allowed for better visualization and more precise surgical maneuvers to be performed within the clinic or minor procedure room. While not a replacement for in-OR 3-port pars-plana vitrectomy, the potential of the office-based vitrector now seems expansive [3,4,10-12,14]. The incidence of reported complications and endophthalmitis following office-based vitrectomy remains largely unknown. The largest combined study to date of 4509 single-port procedures performed using the Intrector places endophthalmitis rates at around 0.17% (62.9% in an office setting, 37.1% in an OR setting) [5]. This data differs from our study’s surgical model where all procedures were three-port and performed in the office setting. Comparatively, modern endophthalmitis rates for standard 23-ga and 25-ga MIVS are reported at around 0.02-0.10%, although values ranging from 0.02%-0.80% have been described throughout the years [9,15-23].

Our study is the first to detail microbial colonization of the ocular surface and corresponding vitreous contamination for office-based portable MIVS. Our preliminary survey suggests that this is a well-tolerated procedure with little long-term complications. Our conjunctival flora culture positivity is slightly lower than that previously reported in vitrectomy literature (46% instead of 61-98%) [8]. This may be due to the difference in our sampling method. To reduce lid contamination, we only cultured the bulbar conjunctiva where the instruments would be introduced. Based on prior literature, we also chose not to administer prophylactic topical antibiotics to patients prior to sampling [6,8]. Despite the lack of topical antibacterial prophylaxis, only 1/68 vitreous samples were culture positive. It is likely that the 5% povidone-iodine preparation left on the surgical field for 5 minutes prior to the start of the case is in part responsible for this finding [6,7,9]. *Shimada et al.* have even advocated for the repeated irrigation of 0.25% povidone-iodine unto the surgical field during a procedure to reduce vitreous contamination. However, as per the authors, the safety and efficacy of such a practice needs further statistical confirmation from a larger scale study necessitating enrollment of over 50,000 eyes [7].

Several studies postulated that compared to the 20-ga vitrector, the smaller lumen of a 25-ga instrument reduces the infusion rate of BSS, hence slowing the “flushing” of the vitreous cavity, making the exit of bacteria more difficult [8,16,17,23]. In the case series presented by Tominaga et al. on the microbiological study comparing 20-ga to 25-ga vitrectomies, 22.5% (9/40) of patients undergoing 25-ga MIVS had vitreous contamination at the beginning of the vitrectomy surgery. This number decreased to 0% at the end of the vitrectomy, with the authors hypothesizing that the constant infusion of BSS during surgery might possibly play a “cleansing” role [8,16,23]. Our own results did not demonstrate such a dramatic decrease in vitreous bacterial load since there was already little bacterial contamination at the beginning of vitrectomy. Perhaps this may be due to the reduced surgical time (on average 10 minutes) and the sturdy hypodermic, needle-type build of the Retrector, manufacturing sharper and cleaner wounds [5].

**Figure 3:**
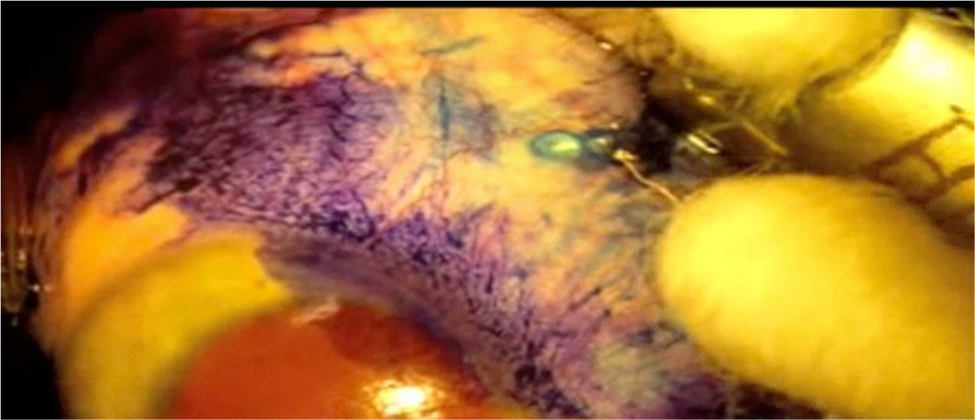
**Vitreous wick.** Trypan blue staining of the conjunctiva highlighting a vitreous wick after removal of the 23-gauge Retrector. If present, the vitreous was cut and removed with the vitrector and conjunctiva was closed with cauterization.

A positive vitreous culture fortunately does not often lead to endophthalmitis. Previous studies in both cataract and vitrectomy literature have shown that in most surgical cases with culture-proven anterior chamber or vitreous contamination, there is no spread of infection. For example, Egger *et al.* studied 25 consecutive subjects undergoing primary 20-ga pars plana vitrectomy: 32% (n = 8) had vitreous contamination at the beginning of vitrectomy surgery, 20% (n = 5) had vitreous contamination towards the end of surgery, yet none developed endophthalmitis postoperatively [19]. More recently in cataract literature, several studies have shown that the approximate rate of anterior chamber contamination at the end of uncomplicated surgery is around 2%, yet the rate of acute postoperative endophthalmitis is below 0.1% [24-26]. Conversely, a negative culture does not equate to a sterile environment and endophthalmitis may still arise. This may be due to two main reasons. First, only a portion of the vitreous is sampled and bacteria present in low numbers may have been missed during collection or that the culture method was not sensitive enough to detect the minute amount of organisms. Second, if the contamination does not arise from the conjunctiva pre- and intra-operatively, it may still occur post-operatively. On several occasions, we observed and documented on high magnification with Trypan blue staining the active prolapse of vitreous content through the sclerotomy after removal of the vitrector needle (Figure 3). If this prolapse remains incarcerated, this would imply the establishment of a patent conduit between the ocular cavity and the external surface in the post-operative period. This led us to pay particular attention to better conjunctival wound closure at the end of each case [9,15-17,22,23].

While the conclusions of our study are thought provoking, some inherent limitations to experimental design include the single surgeon, prospective nature of the current study, limiting extrapolations as to the external validity of this study*.* In addition, the patient cohort is small, with a relatively low bacterial detection rate within both undiluted and diluted vitreous. It is possible that to increase the sensitivity of bacterial detection and to further prove that the same genetic strain of bacteria within the vitreous cavity came from the conjunctival flora, quantitative PCR could be used in addition to conventional culture in future cases [27-30]. Currently, such techniques have only been advocated for cases when minute quantities of bacteria are present, especially once antibacterial or antiviral therapies have been initiated [24,28-31].

## Conclusion

The observed microbiological profiles indicate that although the ocular surface is well inhabited by gram-positive flora, the risks of vitreous bacterial contamination is low during the set-up and process of the surgery. Hence, the usefulness of BSS infusion in vitreous cleansing remains to be further elucidated. We hope that increased experience and attention paid in future large-scale studies to the possible routes of post-operative contamination will streamline approaches and further mitigate the current rates of endophthalmitis in office-based vitrectomies. We hope to witness this evolution make its use easier and more accessible in the future as a safe alternative to conventional vitrectomy for simple and short vitreoretinal surgical cases.

## Competing interests

Flavio Rezende and Przemyslaw Sapieha: Department of Ophthalmology, University of Montreal; Department of Ophthalmology, Maisonneuve-Rosemont Hospital (HMR); Fond de Recherche en Ophtalmologie, University of Montreal (FROUM); Foundation Fighting Blindness Canada (FFB); Retina Foundation of Canada; Insight Instruments, Stuart, FL; Synergetics Inc, O’Fallon MO; Novartis Canada, Montreal, QC.

Cynthia Qian: None.

## Authors’ contributions

Involved in design and conduct of study (FAR, CXQ, PS); Collection of data (FAR, CXQ); Statistical analysis (CXQ); Data interpretation (FAR, CXQ, PS); Preparation, review or approval of the manuscript (FAR, CXQ, PS).

## Authors’ information

Flavio A Rezende and Cynthia X Qian are joint first authors.

## Pre-publication history

The pre-publication history for this paper can be accessed here:

http://www.biomedcentral.com/1471-2415/14/58/prepub
